# Recreating the Bone Marrow Microenvironment to Model Leukemic Stem Cell Quiescence

**DOI:** 10.3389/fcell.2021.662868

**Published:** 2021-09-13

**Authors:** Eimear O’Reilly, Hojjat Alizadeh Zeinabad, Caoimhe Nolan, Jamileh Sefy, Thomas Williams, Marina Tarunina, Diana Hernandez, Yen Choo, Eva Szegezdi

**Affiliations:** ^1^Apoptosis Research Centre, Department of Biochemistry, School of Natural Sciences, National University of Ireland Galway, Galway, Ireland; ^2^Plasticell Ltd., Stevenage Bioscience Catalyst, Stevenage, United Kingdom

**Keywords:** leukemic stem cell, acute myeloid leukemia, bone marrow microenvironment, quiescence, three-dimensional model

## Abstract

The main challenge in the treatment of acute myeloid leukemia (AML) is relapse, as it has no good treatment options and 90% of relapsed patients die as a result. It is now well accepted that relapse is due to a persisting subset of AML cells known as leukemia-initiating cells or leukemic stem cells (LSCs). Hematopoietic stem cells (HSCs) reside in the bone marrow microenvironment (BMM), a specialized niche that coordinates HSC self-renewal, proliferation, and differentiation. HSCs are divided into two types: long-term HSCs (LT-HSCs) and short-term HSCs, where LT-HSCs are typically quiescent and act as a reserve of HSCs. Like LT-HSCs, a quiescent population of LSCs also exist. Like LT-HSCs, quiescent LSCs have low metabolic activity and receive pro-survival signals from the BMM, making them resistant to drugs, and upon discontinuation of therapy, they can become activated and re-establish the disease. Several studies have shown that the activation of quiescent LSCs may sensitize them to cytotoxic drugs. However, it is very difficult to experimentally model the quiescence-inducing BMM. Here we report that culturing AML cells with bone marrow stromal cells, transforming growth factor beta-1 and hypoxia in a three-dimensional system can replicate the quiescence-driving BMM. A quiescent-like state of the AML cells was confirmed by reduced cell proliferation, increased percentage of cells in the G_0_ cell cycle phase and a decrease in absolute cell numbers, expression of markers of quiescence, and reduced metabolic activity. Furthermore, the culture could be established as co-axial microbeads, enabling high-throughput screening, which has been used to identify combination drug treatments that could break BMM-mediated LSC quiescence, enabling the eradication of quiescent LSCs.

## Introduction

Acute myeloid leukemia (AML) is the most common acute leukemia in adults, constituting approximately 80% of all acute leukemia cases diagnosed (Harrington, 2011). Patients are generally treated with the nucleoside analog cytarabine (AraC) in combination with anthracycline antibiotics, such as daunorubicin (Dnr) or idarubicin. While complete remission is achieved in the majority of patients, approximately two-thirds of them relapse (Lee and Muller, 2010), resulting in an overall 4-year survival between 30 and 40% (Hanahan and Weinberg, 2017). Studies have shown that the primary cause of relapse is the presence of a treatment-resistant AML subpopulation known as leukemia-initiating cells or leukemic stem cells (LSCs) (Jonas, 2017).

Leukemic stem cells share many features with their normal counterparts, including existence in a state of quiescence which is characterized by low rate of RNA synthesis (Nakamura-Ishizu et al., 2014), low metabolic activity (Justino De Almeida et al., 2017) and a high expression of anti-apoptotic proteins, such as Bcl-2 (Sharma and Pollyea, 2018), making them resistant to cellular stress and cell death induced by chemotherapeutics. Consequently, LSCs can outcompete normal HSCs or “hijack” the bone marrow microenvironment (BMM) and occupy the HSC niches (Nwajei and Konopleva, 2013).

Studies have shown that quiescent HSCs are protected from myeloablative agents, such as 5-fluorouracil and AraC, as these agents are only effective on cycling cells. Consequently, when quiescent HSCs are forced to enter the cell cycle—for example, by cytokines—they become sensitive to myeloablative agents (Essers et al., 2009). It is thus hypothesized that LSCs may also be sensitized to chemotherapeutics in the same manner. For example, Saito et al. (2010b) have shown that priming LSCs with granulocyte colony-stimulating factor (G-CSF) resulted in their entry into the cell cycle and sensitization to AraC.

Based on these findings, there is a clinical need to identify treatments able to break LSC quiescence so that LSCs can be more effectively targeted with chemotherapeutics in order to reduce the chance of relapse. However, such studies require advanced experimental models which can replicate the quiescence-mediating BMM.

Here we report a hydrogel-based layered co-culture system which incorporates the key BMM-specific quiescence-inducing signals and is able to establish AML quiescence. The quiescent state of AML cells was confirmed by reduced cell proliferation rate, increased percentage of cells in the G_0_ cell cycle phase and a substantially reduced absolute cell number, expression of quiescence markers, such as p21, and reduced metabolic activity. We translated the co-culture system into co-axial microbeads suitable for high-throughput screening. Using these beads, we were able to identify combinations of drugs that can break BMM-mediated AML quiescence, offering the potential to sensitize LSC to chemotherapeutics and eliminating the AML subpopulation responsible for patient relapse.

## Materials and Methods

### Reagents

Two percent sodium alginate was prepared by dissolving sodium alginate (Sigma-Aldrich) in phosphate-buffered saline (PBS) and sterilizing by heating to 80°C in a water bath for 15 min (Leo et al., 1990). Anti-CD150-PE, anti-GRPC5C Alexa Fluor-405, and anti-Ki67-Alexa Fluor 405 antibodies for flow cytometry were purchased from R&D Systems. Anti-CD49f-PE and anti-CD90-VioBlue were purchased from Miltenyi Biotec. Transforming growth factor beta-1 (TGFβ-1) and fibroblast growth factor were obtained from PeproTech. Fms-like tyrosine kinase-3 (Flt-3), G-CSF, stem cell factor (SCF), and interferon alpha were purchased from ImmunoTools. All-*trans* retinoic acid (ATRA) was obtained from Sigma-Aldrich. Hoechst 33342, propidium iodide (PI), and pyronin Y (PY) were purchased from Sigma-Aldrich. The CountBright^TM^ Absolute Counting Beads was purchased from Thermo Fisher Scientific. MHY1485, AZD8055, rosiglitazone, KU-55933, LEE011, roscovitine (seliciclib, CYC202), glasdegib (PF-04449913), sodium butyrate, dasatinib, BIO, plerixafor (AMD3100), quizartinib, and sorafenib were purchased from Selleckchem. Adiponectin, triglitazone LE135, PD169316, harmine, nilotinib, ethylisopropyl amiloride, anisomycin, and curcumin were purchased from Sigma-Aldrich, while prostaglandin E2 was from BioVision/Cambridge Bioscience. Cytarabine and daunorubicin were purchased from Sigma-Aldrich.

### Cell Culture

KG1a cells were maintained at a density of 500,000 cells/ml in RPMI-1640 supplemented with GlutaMAX (Gibco, 2 mM), 10% HyClone fetal bovine serum (FBS, Thermo Fisher Scientific), penicillin (100 U/ml), streptomycin (100 μg/ml), and sodium pyruvate (1 mM). Bone marrow mesenchymal stromal cells (BMSCs; hTERT immortalized primary BMSCs from a healthy donor) were cultured at a density of 50,000 cells/ml in αMEM (Sigma-Aldrich) supplemented with 10% HyClone FBS, penicillin (100 U/ml)/streptomycin (100 μg/ml), sodium pyruvate (1 mM), and GlutaMAX (2 mM).

### Generation of Indirect-Contact Co-culture System

BMSCs were resuspended at 500,000 cells/ml in 2% alginate solution and plated into 24-well plates (200 μl/well). Alginate was crosslinked by adding 100 mM CaCl_2_ for 10 s, forming a layer of BMSCs trapped in the alginate scaffold. Excess CaCl_2_ was removed with three washes of DPBS without Ca^2+^ and Mg^2+^ (Gibco). KG1a cells were labeled with the long-term cell tracker, CFSE (2.5 μM) (see section “Cell Cycle Analyses”), and seeded on top of the alginate-trapped BMSCs in IMDM medium supplemented with TGFβ-1 (10 ng/ml) or ATRA (2.5 μM) at a density of 50,000 cells/ml.

### Immunophenotyping and Viability Assays

The viability of KG1a cells was determined using Annexin V staining. KG1a cells were collected from single cultures by pipetting. From alginate co-cultures, in addition to pipetting off the suspension cells, 200 μl of 1 mM ethylenediaminetetraacetic acid (EDTA) was also added to the top of the alginate to loosen the alginate Annexin V buffer (10 mM HEPES/NaOH, pH 7.5, 140 mM NaCl, 2.5 mM CaCl_2_) containing 1.5 μl Annexin V-APC (ImmunoTools). The cells were incubated on ice in the dark for 15 min, followed by analysis by flow cytometry (BD FACSCanto II, BD Fortessa). Five microliters of CountBright^TM^ Absolute counting beads (Molecular Probes, 0.52 × 10^5^ beads/50 μl) were added to each sample before analysis to enable absolute cell number determination. Data analysis was conducted using FlowJo (V10).

Cell viability in the co-axial beads was assessed using Calcein AM (Molecular Probes). The Calcein AM was diluted 1:500 in the required media. Half of the media from the beads was removed and replaced with an equal amount of the Calcein AM solution (1:1,000 dilution). This was incubated for 30 min at 37°C in the dark. The beads were visualized using fluorescent microscope under the FITC filter (Nikon Eclipse TE2000-S).

For immunophenotyping, cells were harvested and resuspended in 1% bovine serum albumin (BSA)/PBS and blocked for 10 min after which they were collected and resuspended in 100 μl 1% BSA/PBS containing 2 μl of the following antibodies: SLAMF1/CD150-PE (R&D Systems), GRPC5C Alexa Fluor-405 (R&D Systems) CD49f-PE, and CD90-VioBlue (Miltenyi Biotec). The cells were incubated on ice in the dark for 20 min, then washed with 400 μl of 1% BSA/PBS, and resuspended in 200 μl of 1% BSA/PBS for measurement.

### Cell Cycle Analyses

Cell proliferation rate was monitored with CFSE dye retention assay. 5 × 10^6^ KG1a cells were washed twice with 5 ml of Hanks’ solution and the cell pellet was resuspended in 1 ml of Hanks’ solution. CFSE was added to give a final concentration of 2.5 μM and incubated for 20 min at 37°C in the dark. Excess CFSE was removed by washing twice with Hanks’ solution. Finally, the cells were resuspended in full IMDM growth medium to a final concentration of 50,000 cells/ml for seeding. As the CFSE content reduces by half with every division, we were able to extrapolate the drop in the CFSE signal intensity by fitting the fluorescent geometric mean read-outs on a hyperbolic curve, thus estimating the number of cell division cycles that took place (Supplementary Figure S1).

Cells in the G_0_ phase were identified with Hoechst 33342/Pyronin Y staining or with Ki67/PI staining. For Hoechst 33342/Pyronin Y staining, 250,000 KG1a cells were harvested and resuspended in full growth medium, and Hoechst 33342 was added at a final concentration of 20 mg/ml and the cells were incubated in the dark for 45 min at 37°C with intermittent mixing. Pyronin Y (PY) was then added at a final concentration of 0.5 μg/ml, and the cells were incubated for an additional 15 min at 37°C. The samples were then analyzed immediately by flow cytometry.

For Ki67/PI staining, 1 × 10^6^ KG1a cells were harvested and resuspended in 50 μl of PBS. The cells were fixed with 80% ice-cold ethanol (EtOH) and stored at −20°C for a minimum of 2 h. The cells were removed from EtOH and washed twice with 3 ml of PBS. A total of 250,000 cells were then resuspended in 400 μl of blocking buffer (1% BSA/PBS) and incubated for 10 min, followed by incubation in 1% BSA/PBS with Ki67 antibody for 20 min at room temperature (RT) in the dark. Excess antibody was washed off with 1% BSA/PBS, after which the cells were resuspended in the PI staining solution (10 μg/ml PI and 100 μg/ml RNAse A in 1% BSA/PBS) and incubated in the dark at RT for 30 min, with mixing, followed by analysis by flow cytometry.

Cell proliferation in the co-axial beads was assessed with EdU Click-it staining (Thermo Fisher Scientific). The EdU (component A—nucleoside analog) was added to the beads (10 μM) 1 day after the final set of drug treatments had commenced. After 3 days of treatment, the co-axial beads were collected, washed with PBS, and fixed with 4% paraformaldehyde at RT for 30 min. The beads were then incubated in permeabilization solution (0.25% Triton^®^ X-100 in 0.1% BSA in PBS) for 20 min at RT. After washing with 3% BSA in PBS, the beads were re-suspended in Click-iT^®^ Plus reaction cocktail (containing Alexa Fluor 480 picolyl azide) and incubated in the dark for 30 min at 37°C. After the reaction cocktail was removed, the beads were washed and visualized by microscopy (Nikon Eclipse TE2000-S).

### Real-Time Quantitative PCR

Total cellular RNA was isolated using the RNeasy kit (Qiagen). cDNA was generated from 1 μg of RNA using SuperScript^®^ IV First-Strand cDNA Synthesis Reaction kit (Invitrogen). RT-qPCR was performed in triplicate in a 96-well plate. Each reaction consisted of 4 μl of cDNA (diluted 1:10), 5 μl of 2X Brilliant III Ultra-Fast qPCR Master Mix (Agilent Technologies), and 1 μl of 10X PrimeTime qPCR Assay primer–probe (Integrated DNA Technologies). The following primer/probe sets were employed: Hs.PT.58.801316 (ABL1), Hs.PT.58.38531977 (CDK4), Hs.PT.58.344323 (CDK6), Hs.PT.58.40874346.g (CDKN1A/p21), Hs.PT.58.45564663 (CDKN1B/p27), Hs.PT.58.1677181 (CDKN1C/p57), Hs.PT.58.20045500 (CDKN2C/p18), Hs.PT.58.21147259 (GPRC5C), and Hs.PT.58.24703737 (SLAMF1/CD150). The sequences can be found in Supplementary Table S4. RT-qPCR was carried out using the StepOne Plus Real-Time PCR System (Applied Biosciences).

### Western Blotting

Cells were lysed in whole cell lysis buffer (20 mM HEPES, 350 mM NaCl, 1 mM MgCl_2_, 0.5 mM EDTA, 0.1 mM EGTA, and 1% Igepal-630, pH 7.5, with 0.5 mM DTT (Sigma-Aldrich) and Halt Protease inhibitor cocktail (Thermo Fisher Scientific). Proteins (20 μg) were electrophoresed and transferred onto a nitrocellulose membrane (using iBlot^TM^ Gel Transfer and iBlot^TM^ Transfer Stack nitrocellulose, Thermo Fisher Scientific). After blocking, the blots were incubated with rabbit monoclonal antibodies against CDK4 [Cell Signaling Technology (CST)] and p21 (Santa Cruz) and with mouse monoclonal antibodies against CDK6 (CST) and actin (Sigma-Aldrich). For detection, horseradish peroxidase-conjugated secondary antibodies were used (Pierce). The protein bands were visualized with SuperSignal^®^ West Pico Chemiluminescent Substrate (Thermo Fisher Scientific) on X-ray film (Agfa).

### Metabolic Assays

Oxidative phosphorylation and glycolysis were measured with Seahorse XFp analyzer (Agilent). The XFp Miniplate sensor cartridges were hydrated as per the instructions of the manufacturer. The KG1a cells suspended in the XFp assay medium were collected from single cultures or alginate co-cultures as described above and were attached to the wells of Agilent Seahorse XFp Cell Culture Miniplates coated with poly-L-lysine by centrifugation as per the instructions of the manufacturer at a density of 200,000 cells/well in triplicate. The XFp Miniplate was placed in a carrier tray in a non-CO_2_ incubator at 37°C for 30 min to equilibrate the temperature and pH. Analyses were performed both at basal condition as well as after the injection of oligomycin (1.5 μM), FCCP (1 μM), and rotenone plus antimycin A, both at 0.5 μM using the Seahorse XF Cell Mito Stress Test Kit (Agilent).

### Generation of AML-BMSC Co-cultures in Co-axial Beads

The beads were generated with the Var-V1 encapsulator (Nisco Engineering AG) by electrostatic force. The system consisted of two syringes. One syringe contained the KG1a cells resuspended in methylcellulose, and the other syringe contained the bone marrow mesenchymal stromal cells (BMSCs) resuspended in 2% alginate which passed through the inner and outer lumen of the co-axial needle, respectively. Under a constant electric field, these two solutions combined to form droplets of 300–400 μm in diameter at the end of the co-axial needle where they were sprayed into a bath of 100 mM CaCl_2_ (electrospraying) to ensure instant solidification of the beads before the two fluids got mixed. In the CaCl_2_ solution, the alginate became cross-linked, and the co-axial beads were formed. The KG1a cells within the core were seeded at a density of 50,000 cells/ml. The outer shell containing the BMSCs was seeded at 500,000 cells/ml in 2% alginate.

### Tagging, Flow Cytometry, and Analysis of Co-axial Beads

The co-axial beads were labeled with unique tags (intellectual property of Plasticell Ltd.) comprised of 30 unique population-inert fluorescent microspheres, which were connected to the co-axial beads through a multi-layering technique (Tarunina et al., 2016) after each drug treatment. The treatment conditions inducing KG1a cell cycling were selected based on their EdU Click-it incorporation by fluorescent microscopy. The positively selected beads were dissolved with trypsin/EDTA overnight at 37°C. The tags were analyzed by flow cytometry (BD FACSCanto II) and identified using the Ariadne Bioinformatics software (Plasticell Ltd.) to determine the sequential drug treatments that the co-axial beads were exposed to.

## Results

In order to replicate the quiescence-mediating bone marrow microenvironment, we tested: (1) the effect of reduced serum concentration, which has been shown to induce quiescence in multiple cell types (Gos et al., 2005; Li et al., 2019), (2) the co-culture with BMSCs known to secrete key HSC quiescence-inducing cytokines [angiopoietin-1, osteopontin, thrombopoietin, and CXC motif chemokine ligand 12 CXCL12 (data not shown)], (3) retinoic acid (Cabezas-Wallscheid et al., 2017), and (4) TGFβ-1 (Wang et al., 2018), known HSC quiescence-inducing factors within the BMM which are not produced by BMSCs, and (5) the contribution of hypoxia.

As a model of LSCs, the low-differentiation status AML cell line, KG1a, was chosen. Phenotyping by flow cytometry confirmed that KG1a cells have an LSC-like phenotype: Lin^–^/CD38^–^/CD34^+^/CD45RA^+^/CD133^–^/CD49f^+^/CD90^–^ (Supplementary Figure S2). Cell proliferation rate was monitored with a CFSE retention assay, and the average number of cell divisions the culture underwent was calculated as described in section “Materials and Methods” (Supplementary Figure S1).

### The Combination of BMSC and TGFβ-1 Can Induce KG1a Cell Quiescence

The first condition to induce quiescence tested was reduced serum concentration. Although it reduced KG1a proliferation rate, it also led to diminished cell viability over time; therefore, it was not used subsequently (Supplementary Figure S3).

**FIGURE 1:**
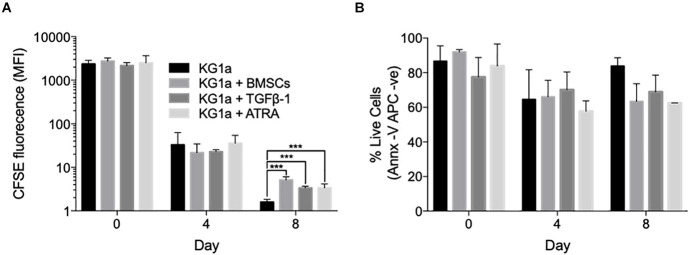
Bone marrow mesenchymal stromal cells (BMSCs), TGFβ-1 and all-*trans* retinoic acid (ATRA) can all reduce KG1a proliferation rate. **(A)** KG1a cells were labeled with 2.5 μM carboxyfluorescein succinimidyl ester (CFSE) and cultured alone, with alginate-encapsulated BMSCs, with TGFβ-1 (10 ng/ml), or with ATRA (2.5 μM) over an 8-day period. The graph shows the mean CFSE fluorescence intensity as a measure of CFSE retention in the live KG1a cells on days 0, 4, and 8 as determined by flow cytometry. **(B)** Viability measured with Annexin V on days 0, 4, and 8 by flow cytometry. ***p < 0.001 (Student’s *t*-test). MFI, mean fluorescence intensity.

To determine the ability of BMSCs to trigger quiescence, a layered, indirect co-culture system was set up by trapping BMSCs in a biocompatible, alginate-based hydrogel, providing a 3D scaffold for BMSCs and seeding the KG1a cells over the hydrogel-embedded BMSCs. Of note, direct co-culture, allowing cell–cell interaction was also tested, but it proved to be unsuitable for long-term culture as the BMSC layer quickly became overgrown and consequently the system deteriorated, indicated by diminishing KG1a cell viability (Supplementary Figure S4). On the contrary, in the indirect, hydrogel-based system, both BMSCs and KG1a cells survived, and the BMSCs showed little to no proliferation, minimizing the risk of BMSC overgrowth during extended culture (Figure 1A and Supplementary Figure S5).

Using the indirect co-culture, the ability of BMSCs to trigger KG1a quiescence was assessed by monitoring CFSE dye retention. Co-culture with BMSCs significantly slowed down KG1a proliferation rate by day 8, with the average division cycle reduced from 7.76 ± 0.01 in a standard culture to 6.65 ± 0.08 cycles in the presence of BMSCs (*p* < 0.001, Welch’s *t*-test; Figure 1A).

TGFβ-1 and retinoic acid are also known as quiescence-inducing factors in the BMM (Cabezas-Wallscheid et al., 2017; Villeval and Vainchenker, 2020). As these factors are not produced by BMSCs, their effect on inducing KG1a cell quiescence was tested by adding them to a KG1a single culture. Both agents reduced the number of division cycles without diminishing KG1a viability (Figure 1B).

**FIGURE 2:**
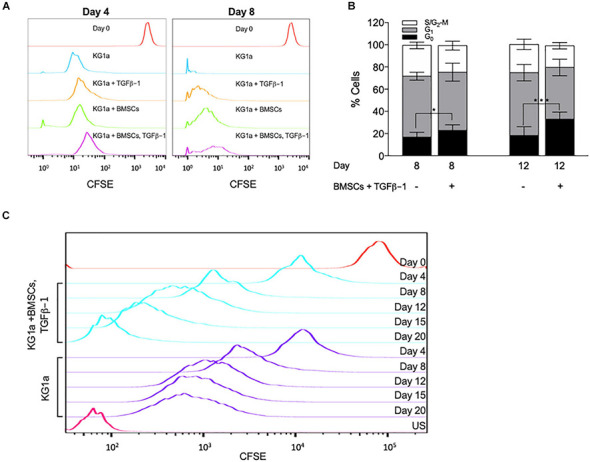
Bone marrow mesenchymal stromal cells (BMSC) and TGFβ-1 have an additive effect on slowing KG1a proliferation and lead to the accumulation of cells in the G_0_ cell cycle phase. KG1a cells were cultured with alginate-embedded BMSC with or without addition of 10 ng/ml TGFβ-1 for the times indicated. **(A)** Histogram representation of carboxyfluorescein succinimidyl ester (CFSE) retention in single culture at day 0 (red) and at day 4 or 8 (blue), culture with TGFβ-1 (orange), BMSC co-culture (green), or both (purple) on days 4 and 8. **(B)** Percentage of cells in the G_0_-, G_1_-, and S/G_2_-M phases of the cell cycle determined by Ki67 + PI staining. **(C)** Histogram representation of CFSE retention in extended single culture (blue) vs. BMSC + TGFβ-1 co-culture (purple). Red, CFSE fluorescence on day 0; US, unstained sample. *p < 0.05; ***p < 0.001 (Student’s *t*-test).

Because the addition of BMSCs, TGFβ-1, and ATRA could all slow down KG1a proliferation, their combined effect was tested. CFSE retention assay showed that BMSCs and TGFβ-1 had an additive effect in reducing KG1a proliferation rate (Figure 2A). ATRA did have a further effect but negatively impacted on KG1a viability (Supplementary Figure S6).

To corroborate these findings, cell cycle distribution analysis was conducted by measuring Ki67 expression combined with DNA content analysis (PI). BMSC + TGFβ-1 increased the percentage of cells in the G_0_ cell cycle phase (Figure 2B). Additionally, by extending the culture to 20 days, CFSE retention showed that, from day 12 onward, the proliferation in the co-culture got almost completely halted, corroborating that the combination of BMSCs and TGFβ-1 induced a state of quiescence (Figure 2C).

### Hypoxia Further Enhances BMSC and TGFβ-1 Induced KG1a Quiescence

**FIGURE 3:**
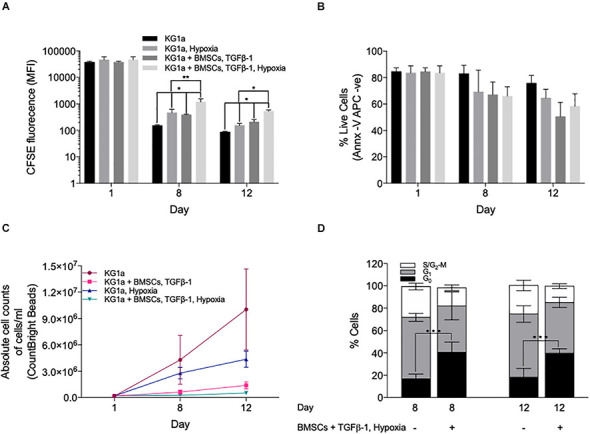
Hypoxia enhances the quiescence-inducing effect of bone marrow mesenchymal stromal cells (BMSCs) and TGFβ-1. KG1a cells were labeled with 2.5 μM carboxyfluorescein succinimidyl ester (CFSE) and cultured alone (KG1a) or with alginate-encapsulated BMSC and TGFβ-1 (10 ng/ml) over a 12-day period in normoxia or hypoxia (1% pO_2_). **(A)** CFSE retention measured in the live cell fraction on days 1, 8, and 12. **(B)** Viability measured with Annexin V on days 1, 8, and 12. **(C)** The absolute number of live cells per milliliter as determined by flow cytometry using CountBright beads on days 1, 8, and 12. **(D)** Cell cycle distribution based on Ki67 expression and DNA content (PI) on days 8 and 12. *p < 0.05; **p < 0.01; ***p < 0.001 (Student’s *t*-test).

Since the hypoxic BM environment (Eliasson and Jönsson, 2010) has also been associated with HSC quiescence (Moirangthem et al., 2015), the ability of a low oxygen concentration (1% O_2_) to trigger KG1a quiescence was tested. Hypoxic conditions significantly enhanced CFSE retention at day 8 (Figure 3A) and enhanced the effect of BMSC + TGFβ-1 without impacting on cell viability (Figure 3B). Absolute cell numbers monitored using counting beads confirmed the reduced cell proliferation rate by over 19-fold in the hypoxic BMSC + TGFβ-1 condition (Figure 3C).

Ki67 + PI staining showed that the reduced proliferation was associated with an accumulation of cells in the G_0_ phase (Figure 3D), which was also confirmed by their reduced RNA content using Pyronin Y staining (Supplementary Figure S7). Collectively, the data shows that co-culture conditions incorporating BMSC, TGFβ-1, and hypoxia have a combined effect in inducing LSC quiescence as seen by dye retention, reducing the absolute cell numbers and the accumulation of cells in the G_0_ cell cycle phase.

To confirm that these culture conditions trigger quiescence, we determined the expression of markers of HSC quiescence, namely, the cell surface marker CD150 (Nakamura-Ishizu et al., 2014; Cabezas-Wallscheid et al., 2017; Tajer et al., 2019) with flow cytometry, GPRC5C, CDK4, and CDK6 (Baker and Reddy, 2012), and the CKI family members p18, p21, p27, and p57 (Cheng et al., 2000; Walkley et al., 2005; Matsumoto et al., 2011; Gao et al., 2015) with quantitative RT-PCR (qRT-PCR). Of note, while CD49f and CD90 are also well recognized markers of HSC quiescence, they were both highly expressed by cycling KG1a cells and thus were deemed to be not suitable markers of KG1a quiescence (Supplementary Figures S1, S8).

**FIGURE 4:**
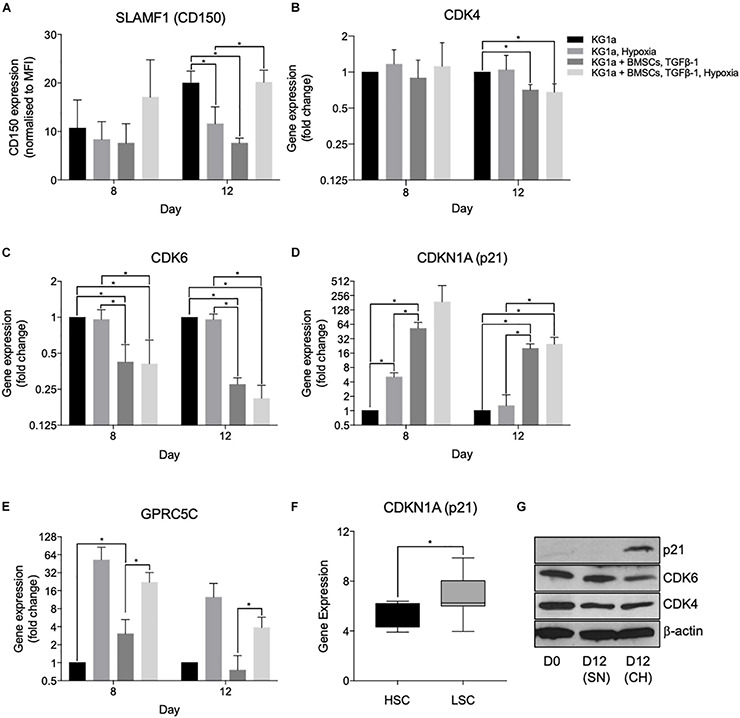
Hypoxia and co-culture with bone marrow mesenchymal stromal cells (BMSC) + TGFβ-1 increases the expression of markers of leukemia cell quiescence. KG1a cells were cultured alone or with alginate-encapsulated BMSC and TGFβ-1 (10 ng/ml) over a 12-day period in normoxia or hypoxia (1% pO_2_). **(A)** Expression of CD150 determined in the live fraction by flow cytometry. MFI, mean fluorescent intensity. *p < 0.05 (Student’s *t*-test). **(B–E)** Results of quantitative RT-PCRs on RNA isolated on days 8 and 12. *p < 0.05 (one-way ANOVA). **(F)** Gene expression analysis of p21 expression conducted on the GSE17054 transcriptomic dataset. The graph represents the changes in gene expression between hematopoietic stem cells and leukemic stem cells. *p < 0.05 (Welch’s *t*-test). **(G)** Western blot images for the expression of p21, CDK4, and CDK6 conducted on samples isolated at day 12 of culture. SN, single culture at normoxia; CH, co-culture under hypoxia.

CD150 was expressed at a low level in cycling KG1a cells, and although we detected an increase in its expression in hypoxia + BMSC + TGFβ-1 culture in comparison to the hypoxic condition alone, there was no significant difference between a normoxic single culture and a hypoxia + BMSC + TGFβ-1 culture (Figure 4A), which was confirmed with qRT-PCR (Supplementary Figure S9).

In the qRT-PCR studies, quiescence-inducing culture conditions repressed the expression of CDK4 and CDK6 and induced the expression of p21 (CDKN1A) and GPRC5C, confirming the induction of quiescence (Figure 4). Reduced CDK6 and induced p21 expression have also been confirmed at the protein level using Western blotting (Figure 4G). Reduced CDK4 protein expression was not detectable, possibly due to the technical limitations of Western blotting, which is not able to show small reductions in protein expression expected based on the level of reduction observed with the qPCR. Interestingly, the expression of p57, the highest expressed CKI in healthy HSCs and other CKIs known to play a role in HSC quiescence (p27 and p18), did not show an increase (Supplementary Figure S9). Overall, the altered expression of these cell cycle proteins further proves the capacity of the developed system to model the quiescence-inducing bone marrow microenvironment. To further investigate that there is a differential dependence of CKIs between HSCs and LSCs, a gene expression analysis was conducted on the open-access transcriptomic analysis of patient-derived LSCs (nine samples) and healthy HSCs (four samples, GSE17054). The expression of p21 alongside p16 and p18 was higher in LSCs in comparison to HSCs (Figure 4F and Supplementary Figures S10A,B), supporting the notion that LSCs utilize different CKIs than HSCs.

**FIGURE 5:**
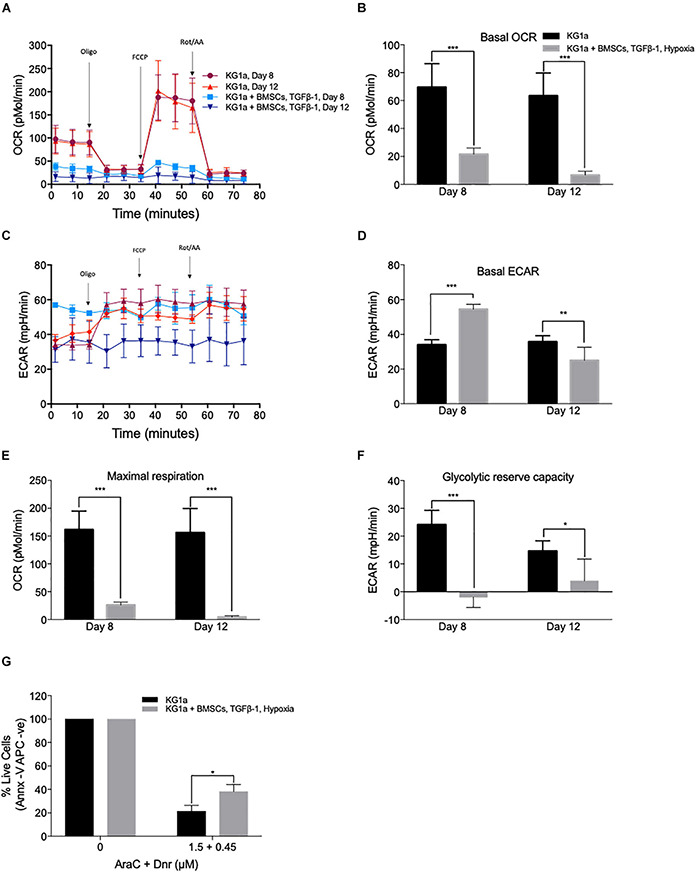
KG1a cells in co-culture have a quiescent leukemic stem cell-like metabolic profile associated with increased drug resistance. KG1a cells were cultured alone in normoxia or with alginate-encapsulated bone marrow mesenchymal stromal cells (BMSC) and TGFβ-1 (10 ng/ml) under hypoxia (1% pO_2_). Oxygen consumption rate (OCR) and extracellular acidification rate (ECAR) were analyzed using the Seahorse XFp analyzer and the Seahorse XF Cell Mito Stress Test kit in samples taken at days 8 and 12. Oligomycin, FCCP, and rotenone/antimycin A were added at the times indicated. OCR **(A)**, basal OCR **(B)**, ECAR **(C)**, basal ECAR **(D)**, maximal respiration **(E)**, and reserved glycolytic capacity **(F)** of KG1a cells alone or in co-culture. **(G)** Co-culture conditions enhance resistance to chemotherapeutics. KG1a cells were cultured alone (KG1a) or in co-culture, as described above, over an 8-day period, followed by exposure to a 3:1 molar ratio of AraC and Dnr for 48 h. The graph represents the percentage of live cells normalized to the untreated control. Viability was measured with Annexin V. *p < 0.05; **p < 0.01; ***p < 0.001 (Student’s *t*-test).

To further investigate how closely the co-culture can replicate the quiescent LSC phenotype, the metabolic profile of the AML cells was studied (Figure 5) using the Seahorse XFp analyzer (Agilent) and the Seahorse XF Cell Mito Stress Test kit. Basal mitochondrial respiration and maximal respiration capacity were measured by determining the mitochondrial oxygen consumption rate in control conditions and after the administration of a proton uncoupler (FCCP). The rate of anaerobic glycolysis and reserved glycolytic capacity were also determined by measuring lactate production (extracellular acidification rate) in control conditions and after blocking the mitochondrial ATP production with the ATP synthase inhibitor, oligomycin. KG1a cells were cultured alone or under conditions of hypoxia + BMSC + TGFβ-1, and measurements were taken at days 8 and 12. KG1a cells in the co-culture displayed a reduced respiration rate (basal OCR) in comparison with the KG1a single cultures (Figures 5A,B). The rate of anaerobic glycolysis was found to be higher in the co-culture on day 8, but it reduced to a low level by day 12 (Figures 5C,D), suggesting that the AML cells in the co-culture stabilize in a quiescent-like state characterized by an overall substantially reduced metabolism, corroborating the proliferation profile, where cell division got almost completely halted by day 12 in the co-culture (Figure 2C). Maximal respiration (Figure 5E) and reserved glycolytic capacity (Figure 5F), two main indicators of the ability of a cell to respond to energy demand upon stress, were also measured. The results showed that the co-cultured cells have a much smaller maximal respiration and reserved glycolytic capacity than the cells in a single culture, which is another known characteristic of drug-resistant quiescent LSCs (Lagadinou et al., 2013).

As quiescence drives drug resistance, the ability of the co-culture system to offer protection against the mainstream AML chemotherapy was tested using the combination treatment of cytarabine + daunorubicin (AraC + Dnr). KG1a cells were cultured alone or under conditions of hypoxia + BMSC + TGFβ-1 for 8 days, after which the cultures were treated with a 3:1 molar ratio of AraC and Dnr [corresponding to the molar ratio of the two drugs used in the clinic (7 + 3 therapy)] for 48 h (Rai et al., 1981). It was found that the co-culture conditions significantly protected the KG1a cells from AraC + Dnr treatment, verifying that the quiescence-inducing co-culture also leads to protection against chemotherapeutics (Figure 5G).

### The Quiescence-Inducing Co-culture System Can Be Translated Into Co-axial Beads Suitable for the Screening of Drugs to Revert Quiescence-Mediated Drug Resistance

In order to test the utility of the developed co-culture system to identify treatments that can re-activate quiescent leukemic cells, the indirect co-culture was developed into a high-throughput screening assay. The BMSC-KG1a co-culture was set up as co-axial beads, where an inner methylcellulose core contained the KG1a cells and an outer alginate shell encapsulated the BMSC. The cells retained their viability in the beads as determined by monitoring their viability with calcein-AM and fluorescence microscopy (Supplementary Figure S5). Since our pilot studies found that single treatments with individual agents had only a marginal effect on re-activating quiescent KG1a cells (Supplementary Figure S11), a sequential treatment matrix consisting of 1,600 different drug combinations was designed based on targeting known pathways that regulate quiescence and the proliferation of HSCs and/or LSC (Supplementary Tables S1, S2). The co-axial beads were exposed to these drug combinations using CombiCult, a platform that enables high-throughput sequential drug tests based on fluorescent labeling and tracking of the beads as they go through the treatment pathways (Supplementary Figure S12).

The co-axial beads were generated and cultured for 4 days prior to drug treatment to induce a slow cycling state, after which they were divided into 10 portions and were subjected to the first set of drug treatments (set 1, Supplementary Table S2) for 3 days. After the treatment, the beads were labeled with a fluorescent tag unique to the treatment that they received (Tarunina et al., 2016), and the beads from the different treatments were pooled together. The pooled beads were then split (resulting in a mixture of beads in each dish representing all possible set 1 treatments) and exposed to the second set of drugs. The labeling, pooling, and splitting process was repeated for the third set of drugs, after which all beads were pooled together, and the effect of the treatments on KG1a cell quiescence was measured with EdU Click-it labeling of cells in the S-phase of the cell cycle.

**FIGURE 6:**
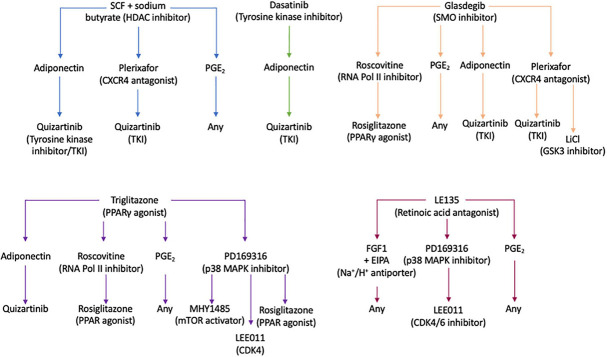
Drug treatment pathways identified by the CombiCult screen to release leukemic stem cells from bone marrow microenvironment-mediated quiescence. The flow charts show the drug treatment sequences which could revert bone marrow mesenchymal stromal cell + TGFβ-1-induced KG1a quiescence. SCF, stem cell factor; CXCR4, CXC motif chemokine receptor type 4; PGE_2_, prostaglandin E_2_; SMO, smoothened; RNA Pol II, RNA polymerase II; PPARγ, peroxisome proliferator-activated receptor gamma; MAPK, mitogen-activated protein kinase; LiCl, lithium chloride; GSK, glycogen synthase kinase 3; mTOR, mammalian target or rapamycin; CDK4/6, cyclin-dependent kinase 4/6.

Using the Ariadne^®^ bioinformatics software (Plasticell Ltd.), treatment sequences which reverted BMSC + TGFβ-1-induced quiescence were identified, and a deconvoluted readout of the drug combinations able to re-activate quiescent KG1a cells was generated (Supplementary Figure S13). From this, the most effective treatment regimens were selected for validation in the layered co-culture system (Figure 6).

**FIGURE 7:**
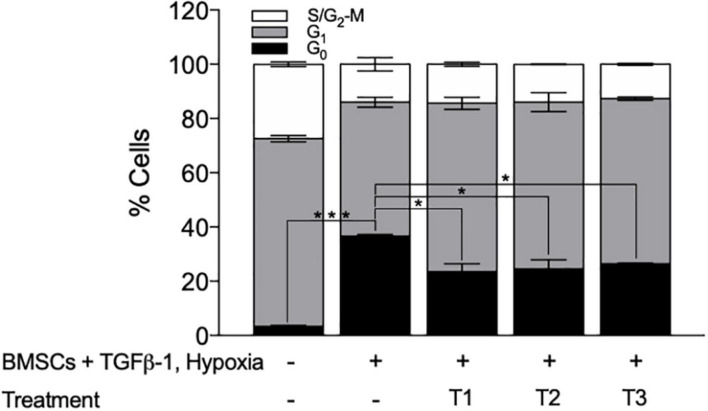
Bone marrow mesenchymal stromal cell (BMSC) + TGFβ-1 + hypoxia co-culture system can be used to identify treatments that can break BMSC–TGFβ-1-induced quiescence. Cell cycle profile of KG1a cells exposed to the drug sequences indicated, measured by Hoechst + PY staining and flow cytometry. T1, glasdegib–adiponectin–quizartinib; T2, glasdegib, plerixafor, quizartinib; T3, stem cell factor + sodium butyrate–adiponectin–quizartinib. *p < 0.05; ***p < 0.001 (Student’s *t*-test).

To validate the screening results, the optimized layered co-culture was employed under conditions of hypoxia. The culture time prior to drug treatment was extended to 8 days to ensure that a quiescent-like state was established. These cultures were then exposed to the three most potent sequential treatments able to reactivate the KG1a cells identified in the CombiCult^®^ screen. The cell cycling rate was monitored with Hoechst + PY staining (identifying quiescent cells based on low RNA content and 2N DNA). The test confirmed that sequential treatments with the hedgehog pathway inhibitor glasdegib-adiponectin (cytokine)-quizartinib (tyrosine kinase inhibitor), glasdegib-plerixafor (CXCR4 inhibitor)-quizartinib, and stem cell factor + sodium butyrate-adiponectin-quizartinib could induce the cycling of quiescent KG1a cells (Figure 7).

Collectively, we have established a layered co-culture system that can faithfully replicate BMM-mediated quiescence. This system was successfully translated into a co-axial bead-based screen used to identify possible drug combinations that could trigger the release of LSCs from quiescence.

## Discussion

Quiescence driven by the BMM is a known mediator of drug resistance in AML (Wang et al., 2017) and it is now well accepted that a subpopulation of quiescent LSCs (or leukemia-initiating cells) resistant to chemotherapeutics is largely responsible for patient relapse (Saito et al., 2010a).

Essers et al. (2009) have shown that HSCs can be released from their quiescent state by interferon alpha (IFNα) and it makes HSCs susceptible to chemotherapeutics. In line with this, Saito et al. (2010b) have shown that pre-treatment with G-CSF triggers entry into the cell cycle and significantly enhances the response of LSCs to AraC. However, to date, there are few experimental models that can replicate the quiescence-inducing BMM *ex vivo* or *in vitro*, which hinders the identification and validation of such treatments. To address this, we developed a system to co-culture KG1a cells (LSC representative) with hydrogel-embedded BMSCs in the presence of TGFβ-1 (Blank and Karlsson, 2015) under conditions of hypoxia. This system induced quiescence in KG1a cells as seen by the increased accumulation of cells in the G_0_ cell cycle phase, reduction in absolute cell number, induction the HSC/LSC quiescence markers GPRC5C and p21, and reduced expression of CDK4 and CDK6 as determined at mRNA and protein levels.

p21 is a member of the Cip/Kip CKI family (CDK2 interacting protein/kinase inhibition protein) together with p27 and p57. In normal LT-HSCs, p57 is the highest expressed member of the Cip/Kip CKI family, and of all CKIs, p57 deficiency shows the strongest developmental defects (Zhang et al., 1997; Takahashi et al., 2000) and depletion of the LT-HSC pool (Matsumoto et al., 2011). Additionally, it has been found that both p57 and p27 drive quiescence of HSCs through interaction with the cyclin D-Hsc70 (constitutive heat shock protein 70) complex, preventing cyclin D1 nuclear translocation and inhibiting the activation of CDK4 and CDK6 (Zou et al., 2011). However, our data shows that p21, rather than p27/p57, is most highly associated with induction of quiescence in LSCs, indicating a potential differential dependence of CKIs upon leukemogenic transformation. There are other studies that support this hypothesis. Tremblay and colleagues found that in T-cell acute lymphoblastic leukemia cell cycle-restricted pre-LSCs express p21, and its deletion can revert drug resistance. The study by Viale et al. (2009) also found that p21 was essential for the maintenance of LSCs by preventing excessive DNA damage and the exhaustion of LSCs.

Another defining characteristics of quiescent LSCs is their low metabolic activity. A state of reduced metabolic activity is likely to support the persistence of LSCs in the BM niche where nutrients are limited and low metabolic activity has also been linked to drug resistance.

In line with this, we found that KG1a cells in co-culture were protected from AraC + Dnr treatment. Additionally, the study by Lagadinou et al. (2013) have shown that drug-resistant LSCs have diminished reserved ATP-producing capacity similar to the AML cells in the quiescence-inducing co-culture in our study. Importantly, they found that this lack of reserved metabolic capacity is a targetable vulnerability of LSCs, and the disruption of mitochondrial oxidative phosphorylation with the Bcl-2 inhibitor, venetoclax, sensitized LSCs to chemotherapeutics (Lagadinou et al., 2013).

By translating the co-culture to co-axial beads, a number of candidate drug combinations able to re-activate quiescent AML cells into cycling were identified. Of these, the combination of SCF with an HDAC inhibitor, such as sodium butyrate, has been employed before to expand HSPCs (Hua et al., 2019). By following this sequential treatment with adiponectin, an adipocyte-derived hormone shown to enhance the exit of HSCs from quiescence (Masamoto et al., 2017) and quizartinib, a tyrosine kinase inhibitor (TKI) that can target c-KIT (Galanis and Levis, 2015) and FLT3 (Naqvi and Ravandi, 2019), this combination could release LSCs from quiescence.

Aberrant Hedgehog signaling has also been identified in a variety of human leukemia types and LSCs. Studies have shown that the transmembrane receptor smoothened (SMO) is involved in the maintenance of LSC quiescence and drug resistance. It has been reported that inhibition of SMO by glasdegib can cause LSCs to re-enter the cell cycle (Sadarangani et al., 2015; Fukushima et al., 2016; Kakiuchi et al., 2017), and an ongoing clinical trial (phase III double-blind, BRIGHT AML 1019) investigates the combination of glasdegib with standard chemotherapy (Cortes et al., 2019) in patients with untreated AML. This corroborates that the developed co-culture model and drug screening platform can be utilized to identify drugs able to release LSCs from quiescence.

Finally, our results also highlight the importance of drug combinations as a treatment strategy. Using mathematical modeling, Glauche et al. (2012) have shown that continuous administration of TKIs with overlapping, short intervals of IFNα as opposed to continuous TKIs plus continuous IFNα or pulsed (single administration) TKI during short intervals of IFNα would be most effective at targeting the leukemic clones in chronic myeloid leukemia. It was hypothesized that the less frequent administration of IFNα may reduce the speed of eradication of leukemic clones but that this approach may prevent the possible exhaustion of normal HSCs (Glauche et al., 2012), thus decreasing possible adverse side effects of combinatorial/sequential treatments.

## Conclusion

The co-culture systems described here can closely model BMM-mediated quiescence and be suitable for high-throughput drug screening. Although it is highly likely that other cells and components of the bone marrow also play a role in inducing quiescence, the presented co-culture model can be a beneficial tool for the identification of treatments and drug targets to reactivate quiescent LSCs and increase their sensitivity to cytotoxic drugs.

## Data Availability Statement

The dataset analyzed (GSE17054) for this study can be found in the Gene Expression Omnibus (GEO) available at https://www.ncbi.nlm.nih.gov/geo/query/acc.cgi?acc=GSE17054.

## Author Contributions

EO’R, ES, and DH designed the study. EO’R and ES wrote the manuscript. EO’R, ES, DH, TW, HZ, and CN preformed the wet-lab experiments. ES and JS preformed the dataset analysis. EO’R, HZ, JS, and ES carried out statistical analyses. EO’R, HZ, CN, JS, MT, TW, DH, YC, and ES contributed to data interpretation and edited and approved the manuscript. All authors contributed to the article and approved the submitted version.

## Conflict of Interest

MT, TW, DH, and YC were employed by Plasticell Ltd., is the owner of the CombiCult^®^ technology. The remaining authors declare that the research was conducted in the absence of any commercial or financial relationships that could be construed as a potential conflict of interest.

## Publisher’s Note

All claims expressed in this article are solely those of the authors and do not necessarily represent those of their affiliated organizations, or those of the publisher, the editors and the reviewers. Any product that may be evaluated in this article, or claim that may be made by its manufacturer, is not guaranteed or endorsed by the publisher.
