# Supplementary material for: Recreating the Bone Marrow Microenvironment to Model Leukemic Stem Cell Quiescence
Source: Front Cell Dev Biol. 2021 Sep 13;9:662868. doi: 10.3389/fcell.2021.662868 (PMC8473680; doi:10.3389/fcell.2021.662868)
Supplement: Supplementary file 1 [file Data_Sheet_1.pdf]

Supplementary Material

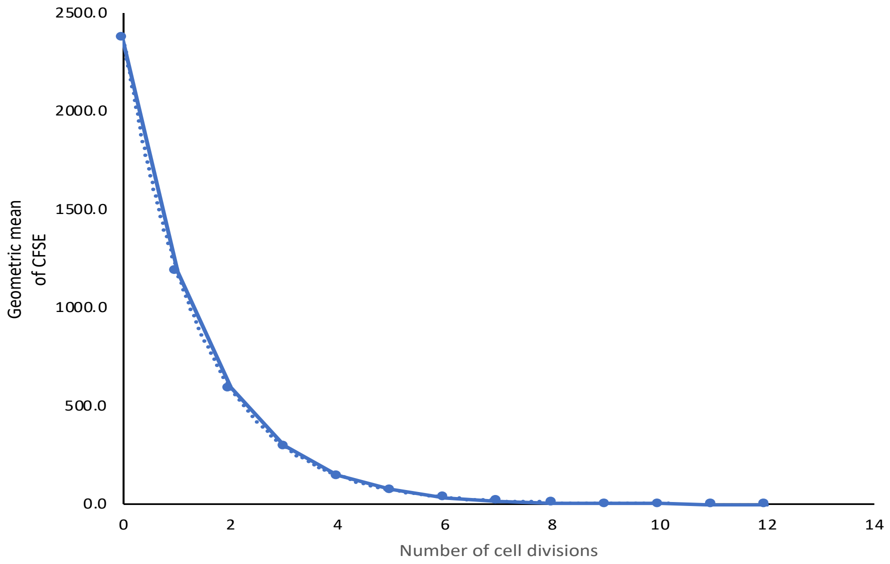

Supplementary Figure 1. Example of hyperbolic curve to extrapolate number of cell divisions. The estimated number of cell divisions that the KG1a cells underwent was determined using a hyperbolic curve whereby initial geometric mean of CFSE on day 0 equated to the CFSE retention of the KG1a cells before undergoing cell divisions. Each time a cell divides, it loses half of its CFSE content, thereby reducing the geometric mean by 2. This was used to generate the hyperbolic curve and allowed the estimated number of divisions to be determined.

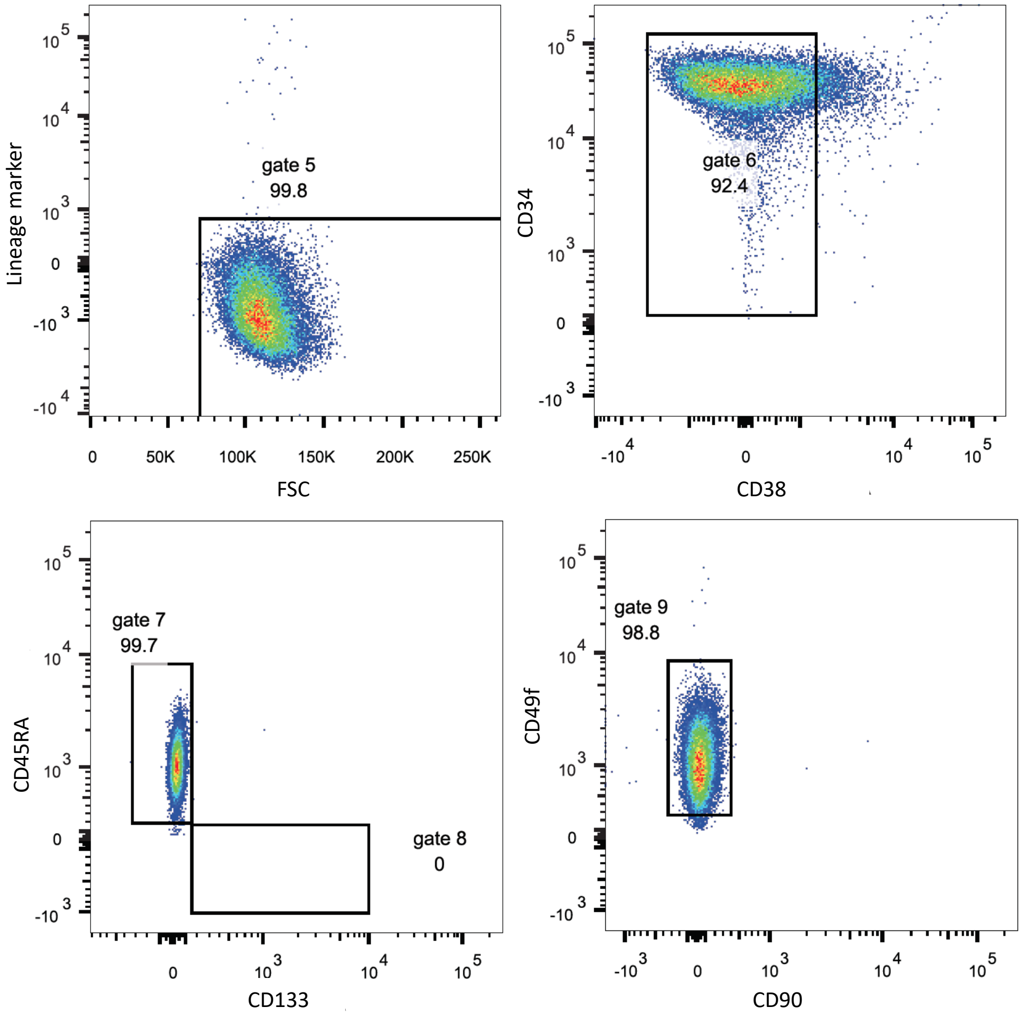

Supplementary Figure 2. Phenotyping of KG1a cells. Flow cytometry analysis of KG1a cells identified that this AML cell line has a primitive phenotype (Lin$^-$, CD34$^+$, CD38$^-$, CD45RA$^-$, CD133$^-$, CD49f$^+$, CD90$^-$).

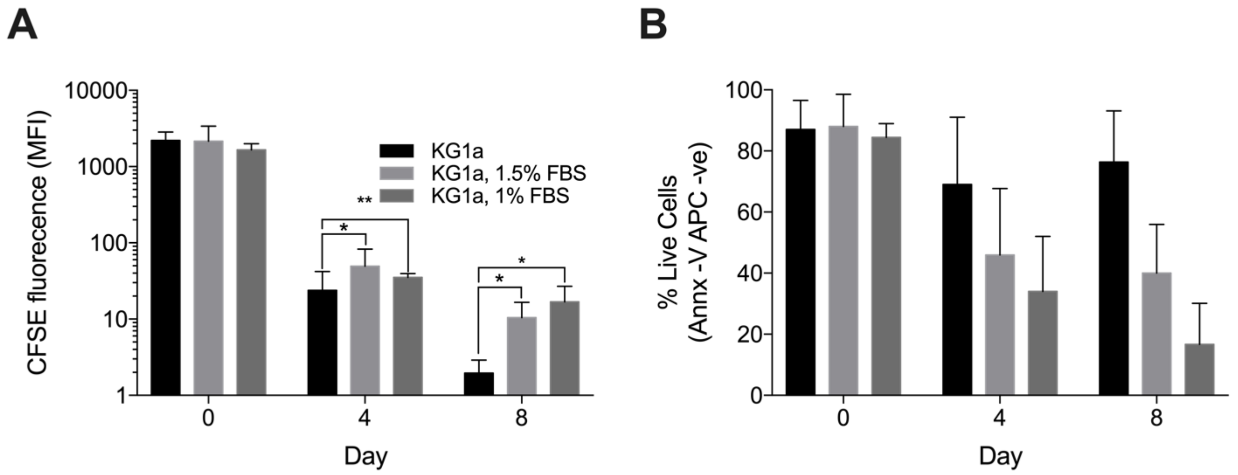

Supplementary Figure 3. Reduced serum concentration can reduce KG1a cell proliferation but compromises viability. (A) CFSE retention measured in the live cell fraction over time with flow cytometry. KG1a cells were stained with 2.5 μM CFSE and cultured over an 8-day period in 10%, 1.5% and 1% FBS-containing medium. (B) Cell viability measured with Annexin V. The graphs show average percentage of live cells ± Stdev from 3 independent repeats. * indicates p <0.05 (student t-test), ** p<0.01. MFI: mean fluorescence intensity.

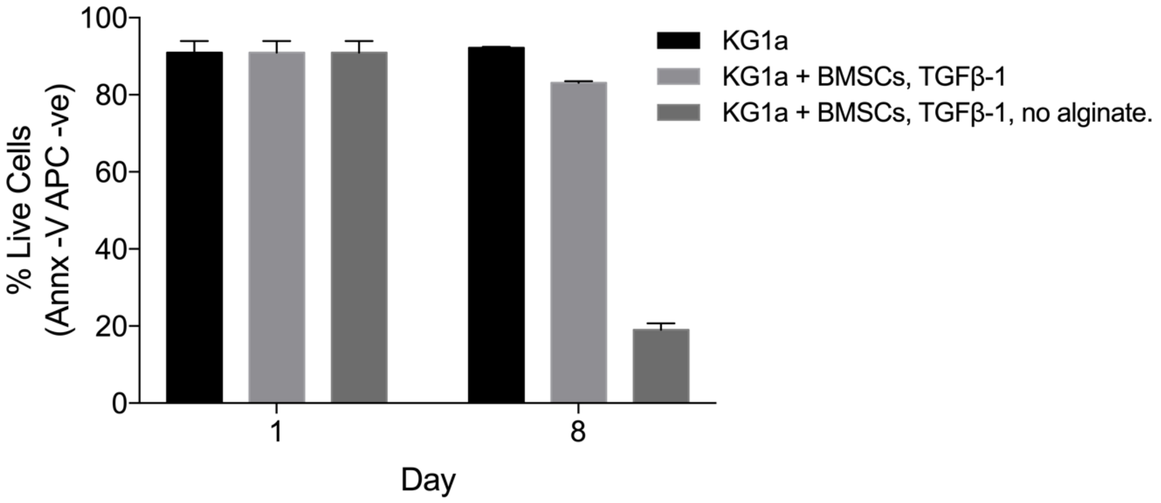

Supplementary Figure 4. 3D alginate co-culture supports the survival of KG1a cells better than a layered system. KG1a cells cultured in alone (KG1a), in direct co-culture with BMSCs + TGFβ-1 or in co-culture with alginate-embedded BMSCs + TGFβ-1 over an 8-day period. Viability measured with Annexin V at day 1 and day 8 by flow cytometry.

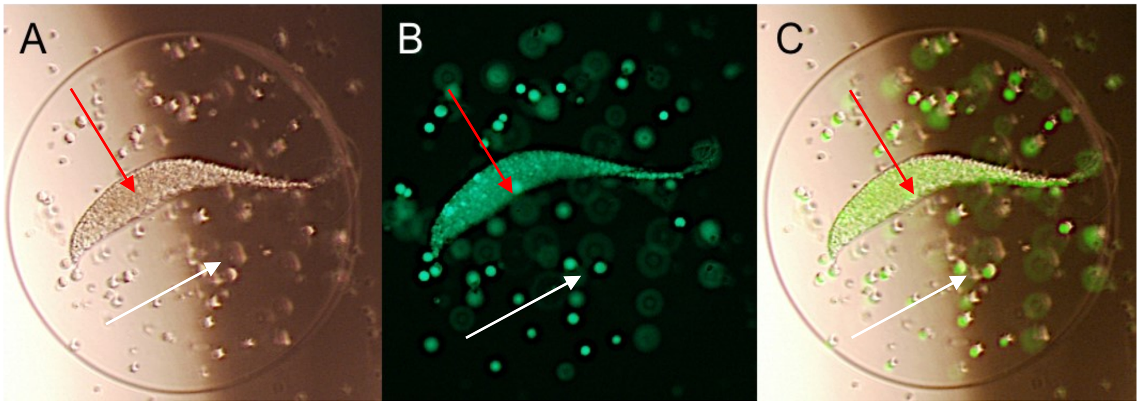

Supplementary Figure 5. Cells in co-axial beads retain viability. Calcein AM staining to assess cell viability in the co-axial beads. (A) Bright field image shows the KG1a inner core (red arrow) and the BMSCs in the outer alginate shell (white arrow), (B) fluorescent microscopy shows cells are viable by Calcein AM. (C) Merging of bright field and fluorescence.

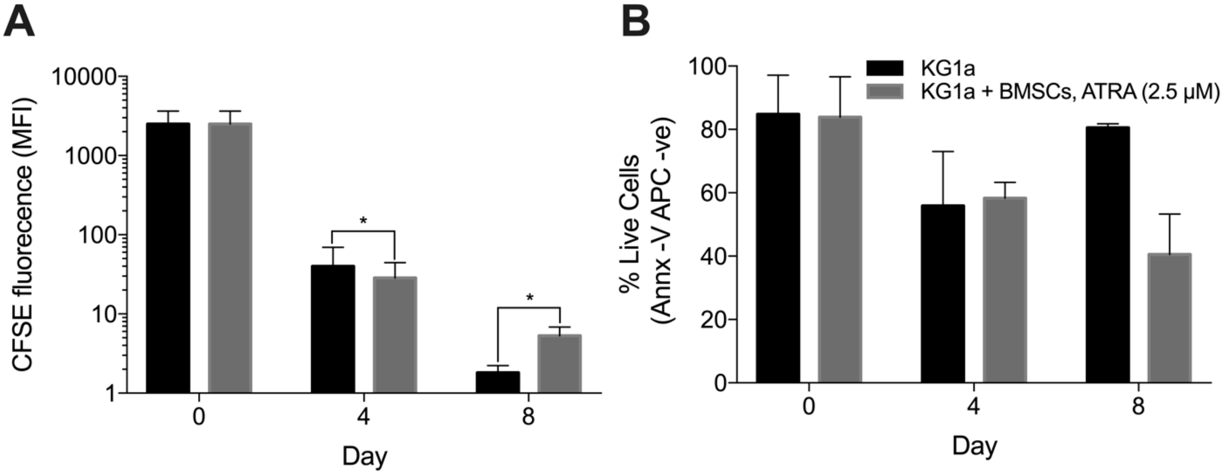

Supplementary Figure 6. The combination of ATRA+BMSCs impairs KG1a cell viability. (A) CFSE retention measured in the live cell fraction over time with flow cytometry. KG1a cells were stained with 2.5 µM CFSE and cultured over an 8-day period. (B) Cell viability measured with Annexin V. The graphs show average percentage of live cells ± Stdev from 2 independent repeats. * indicates p <0.05 (student t-test). MFI: mean fluorescence intensity.

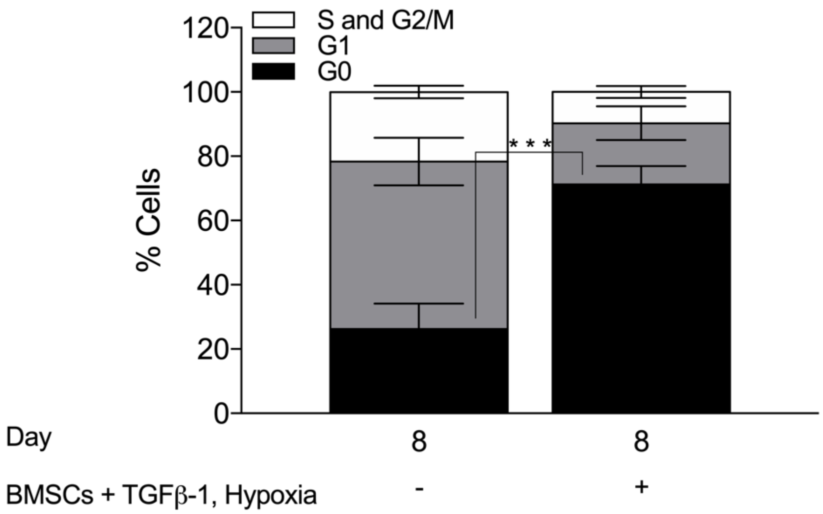

Supplementary Figure 7. Hypoxia enhances the quiescence-inducing effect of BMSCs and TGFβ-1. Cell cycle distribution in KG1a cultures based on dual DNA (Hoechst 33342) and RNA (Pyronin Y) staining on day 8. *** indicates $p < 0.001$ (student t-test).

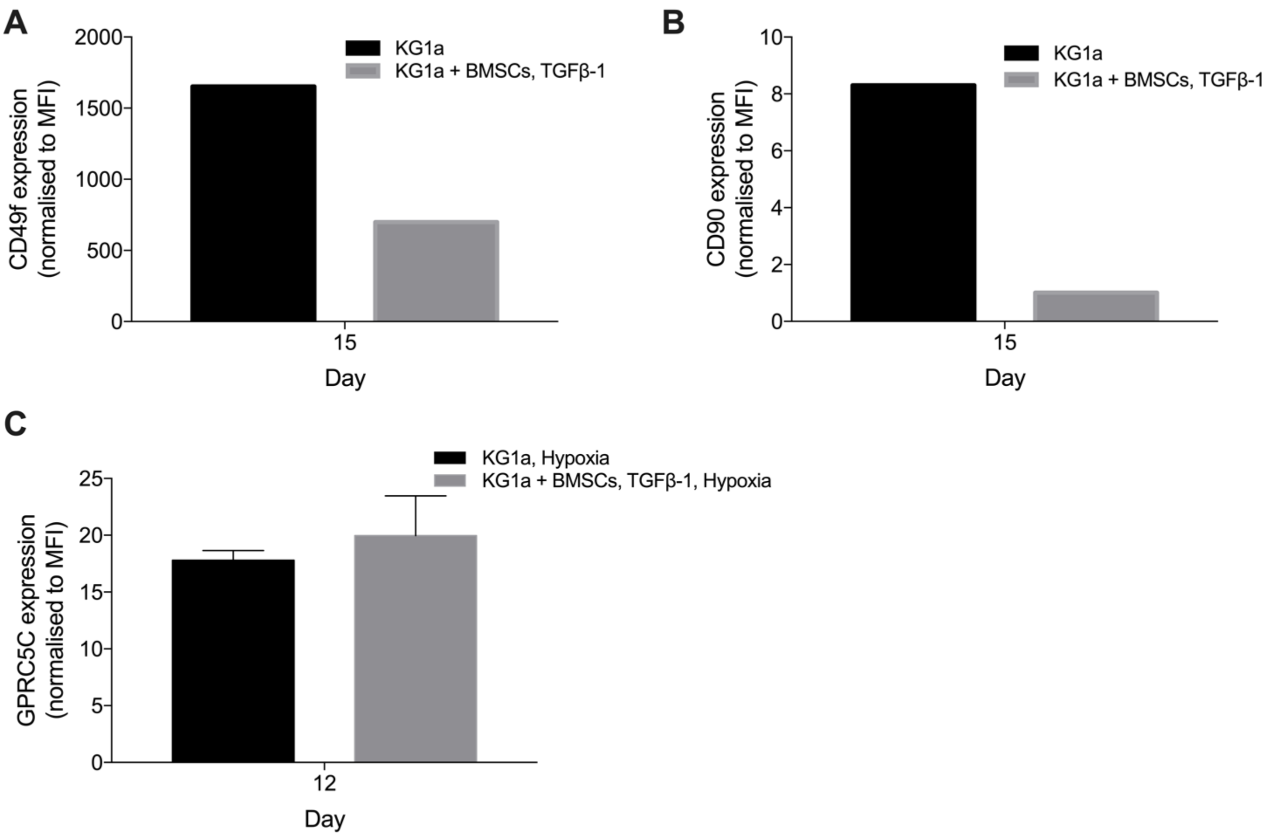

Supplementary Figure 8. Culture with BMSC+TGFβ-1 did not enhance the expression of the quiescence markers CD49f, CD90 and GPRC5C. KG1a cells cultured alone (KG1a) or with alginate-encapsulated BMSCs + TGFβ-1. Viability was measured with Annexin V and expression of CD49f, CD90 and GPRC5C was determined in the live fraction by flow cytometry.

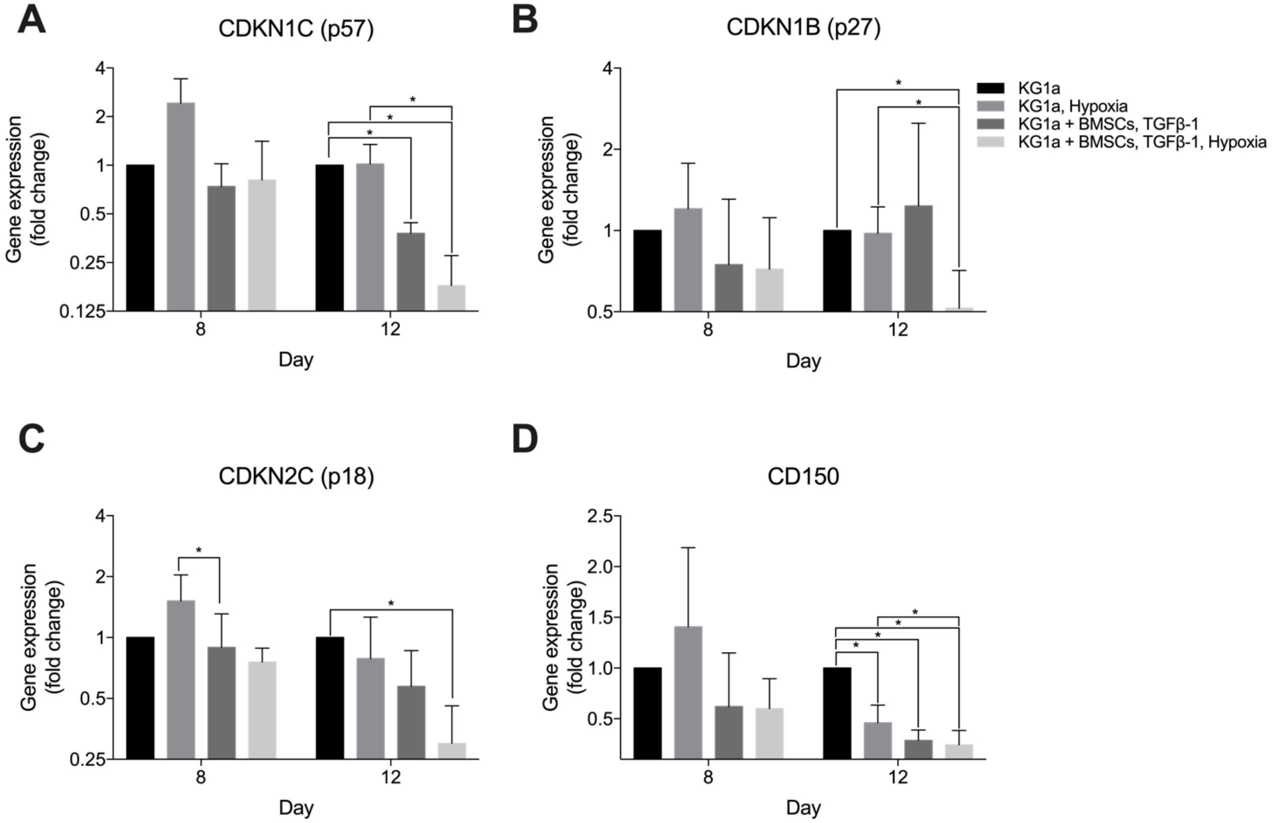

Supplementary Figure 9. Impact of co-culture conditions on the expression of markers of quiescence. Results of quantitative RT-PCRs on RNA isolated on day 8 and day 12. * indicates p <0.05 (one-way ANOVA).

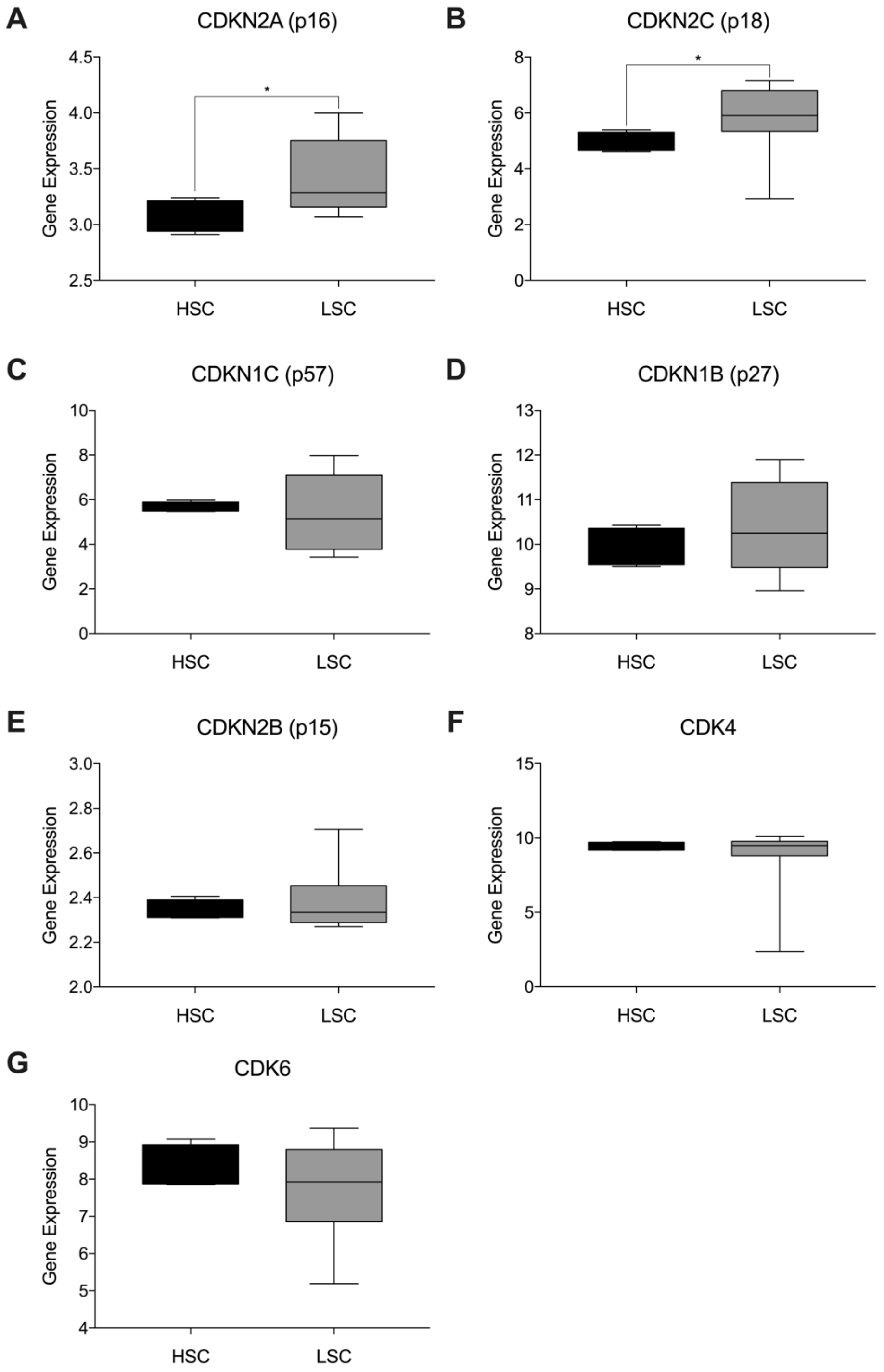

Supplementary Figure 10. Comparison of CKI and CDK gene expression between HSCs and LSCs. Gene expression analysis was conducted on the GSE17054 dataset. The graphs represent the levels of gene expression in HSCs and LSCs. * indicates $p < 0.05$ (Welch t-test).

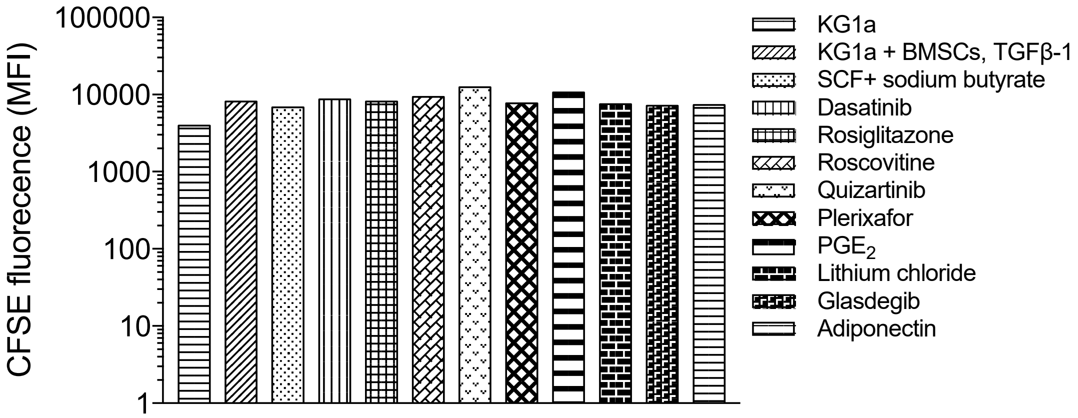

Supplementary Figure 11. Treatment with single agents could not reverse the BMSC+ TGFβ-1 induced quiescence on KG1a cells. KG1a cells were stained with 2.5 μM CFSE and cultured over an 8-day period with BMSCs+TGFβ-1. At day 4, the cultures were treated with the drugs indicated. CFSE retention measured at day 8 in the live cell fraction (Annexin V negative). MFI: mean fluorescence intensity.

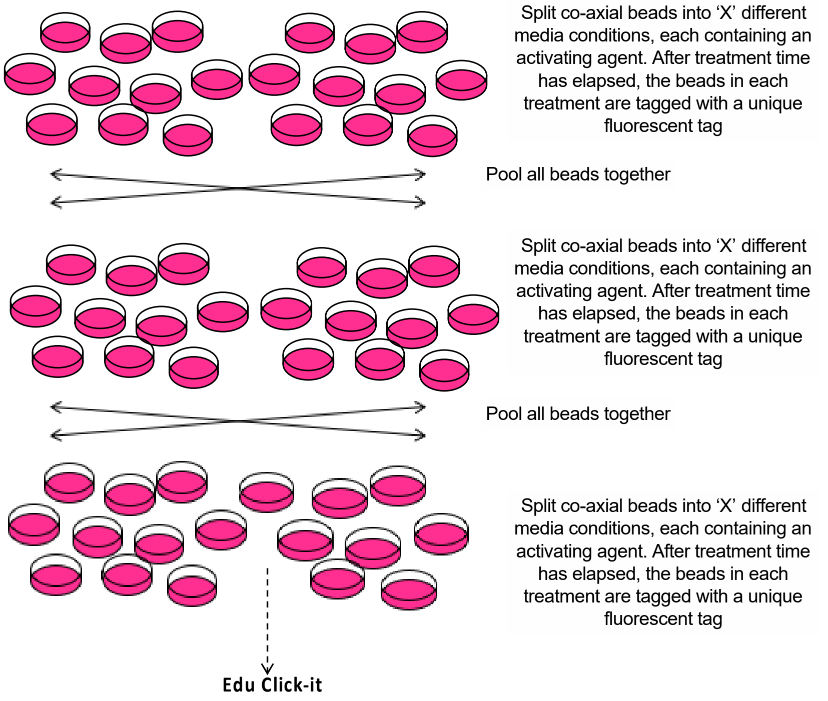

Supplementary Figure 12. Schematic representation of the Combicult screening system. Beads with unique fluorescent tags can be passed through sequential treatment conditions. Re-entry into the cell cycle can be measured by increased cell proliferation (Edu Click-it) and treatment history of the beads can be monitored by fluorescent tags using the Combicult® technology and Ariadne software.

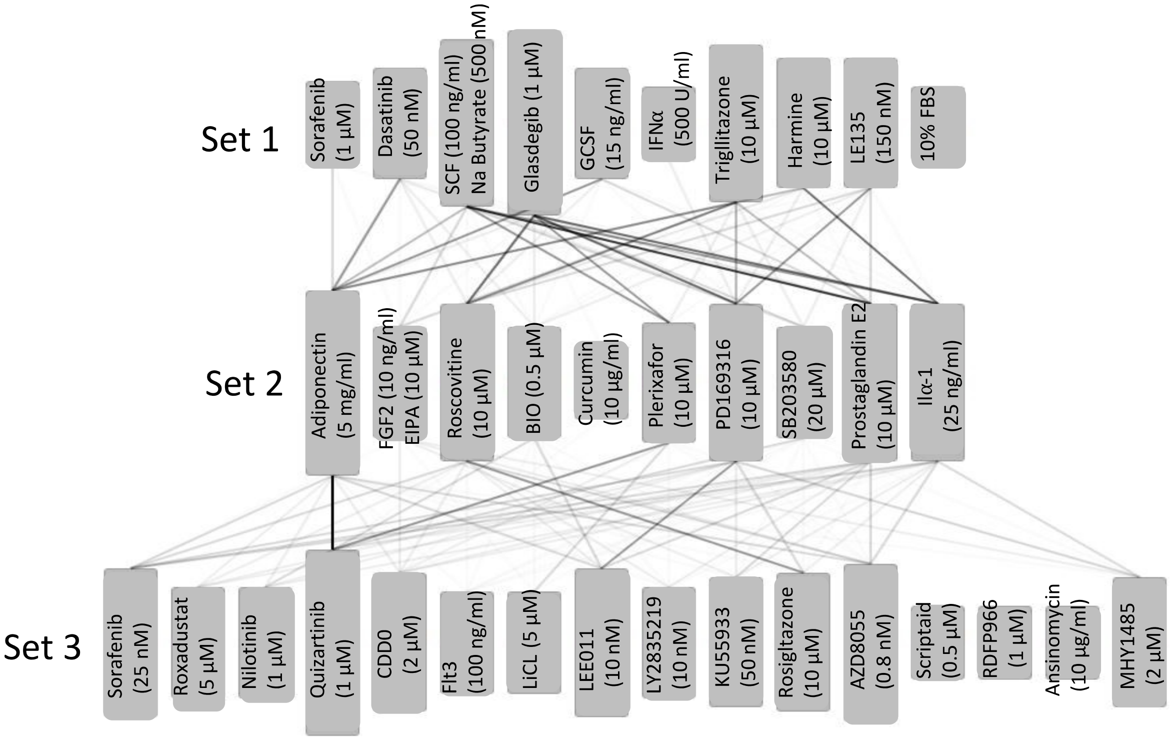

Supplementary Figure 13. The most potent treatment regimens for the reactivation of quiescent KG1a cells determined with the Ariadne software. The above scheme shows the 3 different treatment sets (Set 1, 2 and 3). KG1a cells were treated with set 1 after 4 days in co-culture with BMSCs. After 4 days, each treatment set was tagged with a unique fluorescent tag and the beads were pooled together and subject to set 2. The treatment was conducted for a further 3 days and at the end of set 2, each treatment set was tagged with a unique fluorescent tag and the beads were pooled together and subjected to set 3 for a further 3 days of treatment. Schematic diagram illustrates an overlay of all protocols deconvoluted from 159 hits. The height of boxes representing each cell culture medium is proportional to the number of hits generated by that condition. The opacity of the linkage lines is proportional to the number of hits generated by specific medium combinations–the darkest lines correspond to 8 hits.

Supplementary Table 1. Compounds used in the Combicult screen and their targets.

Set 1	Set 2	Set 3
Sorafenib<br>(Inhibitor of c-KIT/TKI)	Adiponectin<br>(Hormone)	Sorafenib<br>(TKI)
Dasatinib<br>(BCR-Abl, Src family inhibitor)	FGF1 (cytokine) + EIPA<br>(Na+/H+ antiporter)	Roxadustat<br>(HIF-α prolyl hydroxylase inhibitor)
SCF (cytokine) + Sodium Butyrate (HDAC inhibitor)	Roscovitine<br>(RNA Pol II inhibitor)	Niloitinib<br>(Bcr-Abl inhibitor)
Glasdegib<br>(Smoothed Hh inhibitor)	BIO<br>(GSK3 inhibitor)	Quizartinib<br>(c-KIT inhibitor/TKI)
G-CSF<br>(cytokine)	Curcumin<br>(Dietary polyphenol)	CDDO (IKK inhibitor, NF-κB inhibitor)
IFNα<br>(cytokine)	Plerixafor<br>(CXCR4 antagonist)	Flt3 ligand<br>(cytokine)
Triglitazone<br>(PPAR agonist)	PD169316<br>(p38 inhibitor)	Lithium Chloride<br>(GSK3)
Harmine<br>(DYRK1A inhibitor)	SB203580<br>(p38 MAPK inhibitor)	LEE011<br>(CDK4 inhibitor)
LE135<br>(Retinoic acid antagonist)	Prostaglandin E_2 (PGE_2)<br>(Hormone)	LY2835219<br>(CDK4/6 inhibitor)
10% FBS	IL1 α (Cytokine)	KU55933<br>(ATM kinase inhibitor)
		Rosiglitazone<br>(PPAR agonist)

Abbreviations: TKI: tyrosine kinase inhibitor, FGF1: fibroblast growth factor 1, EIPA: Ethylisopropyl amiloride, HIF-α: hypoxia inducible factor-alpha: SCF: stem cell factor, HDAC: histone deacetylase, RNA Pol II: RNA polymerase II, Hh: Hedgehog, GSK3: Glycogen synthase kinase 3, IKK: IκB kinase, NF-κB: nuclear factor kappa B, IFNα: interferon alpha, CXCR4: CXC motif chemokine receptor type 4, Flt3: Fms-like tyrosine kinase 3, PPAR: Peroxisome proliferator-activated receptor, DYRK1A: Dual specificity tyrosine phosphorylation regulated kinase 1A, MAPK: mitogen-activated protein kinase, CDK4/6: Cyclin-dependent kinase 4/6, ATM: ataxia-telangiectasia mutated.

Supplementary Table 2. Drug treatment matrix used for the Combicult® screen.

	**Candidate compounds**									
**Set 1**	SCF (100 ng/ml) [44] + Sodium Butyrate (500 nM) [32]	Dasatinib (50 nM) [45]	IFN-α (500 U/ml) [46]	G-CSF (15 ng/ml) [47]	Triglitazone (10 µM) [48]	Sorafenib (1 µM) [49]	LE135 (150 nM) [12]	Glasdegib (1 µM) [36]	Harmine (10 µM) [50,51]	10% FBS
**Set 2**	FGF-1 (10 ng/ml) + EIPA (10 µM) [52–54]	Roscovitine (10 µM) [55]	BIO (0.5 µM) [56]	Curcumin (10 µg/ml) [57]	Plerixafor (1 µM) [58]	Adioponectin (5 ng/ml) [33]	IL-1α (25 ng/ml) [59]	PGE$_2$ (10µM) [60]	PD169316 (10 µM) [61]	SB203580 (20 µM) [61]
**Set 3**	Sorafenib (25 nM) [49]	Roxadustat (5 µM) [62]	Nilotinib (1 µM) [63]	Quizartinib (1 µM) [34]	CDDO (2 µM) [64]	Lithium chloride (5 µM) [65]	Rosiglitazone (10 µM) [66]		AZD8055 (0.8 nM) [67]	
	LEE011 (10 nM) [70]	LY2835219 (10 nM) [71]	KU55933 (50 nM) [72]	Flt-3 (100 ng/ml) [73]	MHY1485 (2 µM) [33,74]	Scriptaid (0.5 µM) [32]	RGFP966 (1 µM) [68]		Ansinomycin (10 mg/ml) [69]	

Supplementary Table 3. Sequential drug treatments tested on the layered co-culture system

Drug One	Drug Two	Drug Three
Glasdegib	Adiponectin	Quizartinib
Glasdegib	Plerixafor	Quizartinib
Glasdegib	Plerixafor	Lithium Chloride
Glasdegib	Roscovitine	Rosiglitazone
SCF + sodium butyrate	Prostaglandin E_2 (PGE_2)	Quizartinib
SCF + sodium butyrate	Prostaglandin E_2 (PGE_2)	CDDO
SCF + sodium butyrate	Adiponectin	Quizartinib
Triglitazone	Prostaglandin E_2 (PGE_2)	Quizartinib
Triglitazone	Adiponectin	Quizartinib
Dasatinib	Adiponectin	Quizartinib

Supplementary Table 4. Primer and probe sequences

Gene name	Sequence
ABL-1<br><br>(Hs.PT.58.801316)	*Contains:* *nmoles* *Sequence*<br>*Probe* 0.5 **5'-/56-FAM/AGCAATACT/ZEN/CCAAATGCCCAGACGT/3IABkFQ/-3'**<br>*Primer 1* 1.0 **5'-GCCTACAACAAGTTCTCCATCA-3'**<br>*Primer 2* 1.0 **5'-TCTCTAGCAGCTCATACACCT-3'**
CDKN1A/p21<br><br>(Hs.PT.58.40874346.g)	*Contains:* *nmoles* *Sequence*<br>*Probe* 0.5 **5'-/56-FAM/TTCCTCTTG/ZEN/GAGAAGATCAGCCGG/3IABkFQ/-3'**<br>*Primer 1* 1.0 **5'-GCAGACCAGCATGACAGAT-3'**<br>*Primer 2* 1.0 **5'-GAGACTAAGGCAGAAGATGTAGAG-3'**
CDKN1B/p27<br><br>(Hs.PT.58.45564663)	*Contains:* *nmoles* *Sequence*<br>*Probe* 0.5 **5'-/56-FAM/TGTTTCCTT/ZEN/GTTTATCAGATACATCACTGCTTGA/3IABkFQ/-3'**<br>*Primer 1* 1.0 **5'-CAGACGCCCAAGAAGCC-3'**<br>*Primer 2* 1.0 **5'-AAATTTTCATGTATATCTTCCTTGCTTC-3'**
CDKN1C/p57<br><br>(Hs.PT.58.1677181)	*Contains:* *nmoles* *Sequence*<br>*Probe* 0.5 **5'-/56-FAM/CGAAGAAAT/ZEN/CGGAGATCAGAGGCCC/3IABkFQ/-3'**<br>*Primer 1* 1.0 **5'-CGGCGATCAAGAAGCTGT-3'**<br>*Primer 2* 1.0 **5'-GCTTGGAGAGGGACACG-3'**
CDKN2C/p18<br><br>(Hs.PT.58.20045500)	*Contains:* *nmoles* *Sequence*<br>*Probe* 0.5 **5'-/56-FAM/TCCAAGTTT/ZEN/CATAACCTGCAGCGC/3IABkFQ/-3'**<br>*Primer 1* 1.0 **5'-GCACAAAATGGATTTGGAAGGA-3'**<br>*Primer 2* 1.0 **5'-ACCTCTAAGTAGCAGTCTCCTG-3'**
CDK6<br><br>(Hs.PT.58.344323)	*Contains:* *nmoles* *Sequence*<br>*Probe* 0.5 **5'-/56-FAM/CAGCCAACA/ZEN/CTCCAGAGATCCACG/3IABkFQ/-3'**<br>*Primer 1* 1.0 **5'-CGAAGTCTTGCTCCAGTCC-3'**<br>*Primer 2* 1.0 **5'-TCAACATCTGAACTTCCACGA-3'**
CDK4<br><br>(Hs.PT.58.38531977)	*Contains:* *nmoles* *Sequence*<br>*Probe* 0.5 **5'-/56-FAM/AGCCAACAC/ZEN/TCCACATGTCCACA/3IABkFQ/-3'**<br>*Primer 1* 1.0 **5'-TACCGAGCTCCCGAAGT-3'**<br>*Primer 2* 1.0 **5'-TTCAGAGTTTCCACAGAAGAGAG-3'**
CD150/SLAMF1<br><br>(Hs.PT.58.24703737)	*Contains:* *nmoles* *Sequence*<br>*Probe* 0.5 **5'-/56-FAM/AGCCTTACG/ZEN/ATCTATGCCCAAGTCC/3IABkFQ/-3'**<br>*Primer 1* 1.0 **5'-ACGAACCATTACCAGACAACAG-3'**<br>*Primer 2* 1.0 **5'-GGAAGGAGTCAAGTTTCTTCTGA-3'**

GPRC5C<br><br>(Hs.PT.58.21147259)	*Contains:*<br>*Probe*<br>*Primer 1*<br>*Primer 2*	*nmoles*<br>0.5<br>1.0<br>1.0	*Sequence*<br>**5'-/56-FAM/ACTGAGATG/ZEN/GCCCTGATGCACAA/3IABkFQ/-3'**<br>**5'-CATACAGCGGGTACAATGGG-3'**<br>**5'-GGAGGATGATGTCGTAAGCTC-3'**
